# Suppressive Effects of Irbesartan on Inflammation and Apoptosis in Atherosclerotic Plaques of apoE^−/−^ Mice: Molecular Imaging with ^14^C-FDG and ^99m^Tc-Annexin A5

**DOI:** 10.1371/journal.pone.0089338

**Published:** 2014-02-19

**Authors:** Yan Zhao, Ayahisa Watanabe, Songji Zhao, Tatsuo Kobayashi, Keita Fukao, Yoshikazu Tanaka, Toru Nakano, Tetsuya Yoshida, Hiroshi Takemoto, Nagara Tamaki, Yuji Kuge

**Affiliations:** 1 Department of Nuclear Medicine, Graduate School of Medicine, Hokkaido University, Sapporo, Japan; 2 Department of Integrated Molecular Imaging, Graduate School of Medicine, Hokkaido University, Sapporo, Japan; 3 Shionogi Innovation Center for Drug Discovery, Shionogi & Co., Ltd., Sapporo, Japan; 4 Department of Tracer Kinetics & Bioanalysis, Graduate School of Medicine, Hokkaido University, Sapporo, Japan; 5 Central Institute of Isotope Science, Hokkaido University, Sapporo, Japan; King's College London School of Medicine, United Kingdom

## Abstract

**Objectives:**

To investigate the effects of irbesartan on inflammation and apoptosis in atherosclerotic plaques by histochemical examination and molecular imaging using ^14^C-FDG and ^99m^Tc-annexin A5.

**Background:**

Irbesartan has a peroxisome proliferator-activated receptor gamma (PPARγ) activation property in addition to its ability to block the AT1 receptor. Accordingly, irbesartan may exert further anti-inflammatory and anti-apoptotic effects in atherosclerotic plaques. However, such effects of irbesartan have not been fully investigated. Molecular imaging using ^18^F-FDG and ^99m^Tc-annexin A5 is useful for evaluating inflammation and apoptosis in atherosclerotic plaques.

**Methods:**

Female apoE^−/−^ mice were treated with irbesartan-mixed (50 mg/kg/day) or irbesartan-free (control) diet for 12 weeks (n = 11/group). One week after the treatment, the mice were co-injected with ^14^C-FDG and ^99m^Tc-annexin A5, and cryostat sections of the aortic root were prepared. Histochemical examination with Movat's pentachrome (plaque size), Oil Red O (lipid deposition), Mac-2 (macrophage infiltration), and TUNEL (apoptosis) stainings were performed. Dual-tracer autoradiography was carried out to evaluate the levels of ^14^C-FDG and ^99m^Tc-annexin A5 in plaques (%ID×kg). In vitro experiments were performed to investigate the mechanism underlying the effects.

**Results:**

Histological examination indicated that irbesartan treatment significantly reduced plaque size (to 56.4%±11.1% of control), intra-plaque lipid deposition (53.6%±20.2%) and macrophage infiltration (61.9%±20.8%) levels, and the number of apoptotic cells (14.5%±16.6%). ^14^C-FDG (43.0%±18.6%) and ^99m^Tc-annexin A5 levels (45.9%±16.8%) were also significantly reduced by irbesartan treatment. Irbesartan significantly suppressed MCP-1 mRNA expression in TNF-α stimulated THP-1 monocytes (64.8%±8.4% of un-treated cells). PPARγ activation was observed in cells treated with irbesartan (134%±36% at 3 µM to 3329%±218% at 81 µM) by a PPARγ reporter assay system.

**Conclusions:**

Remissions of inflammation and apoptosis as potential therapeutic effects of irbesartan on atherosclerosis were observed. The usefulness of molecular imaging using ^18^F-FDG and ^99m^Tc-annexin A5 for evaluating the therapeutic effects of irbesartan on atherosclerosis was also suggested.

## Introduction

Atherosclerosis is a chronic inflammatory disease in blood vessels that is related to the renin-angiotensin system [1]. Through the angiotensin II type 1 (AT1) receptor, angiotensin II (Ang II) promotes endothelial dysfunction, induces inflammation, and stimulates the oxidation of plasma lipoproteins in atherosclerotic plaques [2], [3]. Since the endothelial dysfunction denotes the initiation of atherosclerosis, enhanced inflammation promotes the development of vulnerable plaques, and reactive oxygen species (ROS) exert harmful effects such as the induction of the apoptosis of macrophage and smooth muscle cells [4], [5], the blockade of the AT1 receptor may suppress atherosclerosis progression and stabilize vulnerable plaques. In agreement with this concern, several experimental studies and clinical trials demonstrated that treatment with angiotensin II AT1 receptor blockers (ARBs) can attenuate atherosclerotic plaque formation, reduce cytokine expression and inflammation levels [6], and suppress oxidative stress in the vessel wall [7].

Irbesartan, one of the most widely used ARBs, has been suggested as a peroxisome proliferator-activated receptor gamma (PPARγ) ligand in addition to its role in the blockade of the AT1 receptor [8]. Since PPARγ activation also exerts anti-inflammatory effects and reduces the ROS production [9], [10], irbesartan may further reduce inflammatory chemokine expression level and suppress apoptotic cell death in atherosclerotic plaque. The anti-atherogenic effects of irbesartan, however, have not been fully investigated, and the mechanisms underlying the therapeutic effects remain unclear.

Although the beneficial effects of irbesartan can be confirmed by the pathological examination of samples collected after surgery or by the indirect assessment of patient outcomes in clinical settings [6], [7], it is also important to non-invasively assess key factors such as inflammation and apoptosis for evaluating the therapeutic effects of irbesartan. Molecular imaging technologies using ^18^F-FDG, a marker of the increased metabolism of inflammatory cells, and ^99m^Tc-annexin A5, a marker of ongoing apoptosis, are logically considered useful in assessing the therapeutic effects on inflammation and apoptosis in atherosclerotic plaques [11], [12]. Accordingly, the anti-inflammatory and anti-apoptotic effects of irbesartan may also be suitably evaluated by radiotracer imaging using the two agents

Therefore, in this study, we aimed to examine the anti-inflammatory and anti-apoptotic effects of irbesartan in a model of spontaneous atherosclerosis (apolipoprotein E knockout mice) with radiotracer imaging as well as histological examination, and to clarify the underlying mechanisms by in vitro experiments.

## Materials and Methods

### Ethical statement

The animal research protocol was approved by the Hokkaido University School of Medicine Animal Care and Use Committee (permit number 08-0061), which also conformed to the Guide for the Care and Use of Laboratory Animals published by the U.S. National Institutes of Health. All efforts were made to minimize suffering.

### Pharmaceuticals, labeled compounds, and reagents

Irbesartan was obtained from Sanofi (Paris, France) or produced in Shionogi & Co., Ltd. Valsartan was purchased from LKB Laboratories Inc. (St. Paul, Minnesota, USA). Universally labeled [^14^C] 2-Fluoro-2-deoxy-D-glucose (^14^C-FDG; specific activity, 11.1 GBq/mmol) in sterile saline was purchased from American Radiolabeled Chemicals, Inc. Recombinant human (rh)-annexin A5 derivatized with hydrazinonicotinamide (HYNIC) was kindly donated by the National Cancer Institute (NCI-Frederick Cancer Research and Development Center, Frederick, MD). HYNIC-annexin A5 was labeled with ^99m^Tc using tricine as the coligand as described previously (specific activity, 4.40±0.13 MBq/µg protein) [13]. All other chemicals used were of the best grade available from commercial sources.

### Animal studies

Studies were performed on female apolipoprotein E knockout (apoE^−/−^, C57BL/6J) mice obtained from Aburahi Laboratories, Shionogi & Co., Ltd. After 9 weeks of age, the mice were weighed and divided into the irbesartan-treated and control groups. The mice in the irbesartan-treated group (n = 11) were fed with a regular research diet (CE-7, Oriental Yeast Co., Ltd.) containing irbesartan. The dose of irbesartan was set at 50 mg/kg/day to correspond to the dose of irbesartan used in humans [14]. After the 12-week treatment, the mice in the irbesartan-treated group were maintained on an irbesartan-free diet for one week before being used in the radio-labeled tracer study. The control mice were fed with an irbesartan-free regular research diet (n = 11) throughout the experimental period.

At 22 weeks of age, the animals were fasted for 12 hr and anesthetized with pentobarbital (50 mg/kg body weight, intraperitoneally). ^14^C-FDG (0.37 MBq/mouse) and ^99m^Tc-annexin A5 (19.41±1.20 MBq/mouse) were intravenously injected into each mouse as a 0.2-ml mixture. Blood glucose level was measured before the tracer injection. Two hours after the ^14^C-FDG and ^99m^Tc-annexin A5 co-injection, the mice were sacrificed by exsanguination under deep pentobarbital anesthesia and their aortas were fixed by cardiac perfusion with cold 0.1 M phosphate-buffered solution (pH 7.4), followed by a cold fixative [4% paraformaldehyde, 0.1 M phosphate-buffered solution (pH 7.4)] [12]. The aortic root was dissected from each aorta, embedded in Tissue-Tek medium (Sakura Finetechnical Co., Ltd.), and frozen in isopentane/dry ice. Serial cross sections of 10 µm (for autoradiographic exposure) or 5 µm (for histochemical staining) thickness were immediately cut and thaw-mounted on glass slides. A total of 264 slices of cross section were analyzed [i.e., 12 slices per animal×(11 irbesartan treated mice+11 control mice)]. Regarding the details, for each animal, four serial slices of the aortic root were examined by autoradiography and eight adjacent slices were randomly assigned to one of the four histochemical staining methods (i.e., Movat's pentachrome staining, Oil Red O staining, Mac-2 staining and TUNEL staining). The average value in each examination for each animal was calculated and used in the analysis.

### Histochemical studies

Movat's pentachrome staining and Oil Red O staining [15] of serial tissue sections were performed. Immunohistochemical staining with a mouse macrophage-specific antibody (Mac-2, clone m3/38, Cedarlane, Ontario, Canada) was also performed in accordance with a standard immunohistochemical procedure [16], with slight modifications as previously described [12]. Apoptotic cells in atherosclerotic plaques were detected using terminal deoxyribonucleotide transferase (TdT)-mediated nick-end labeling (TUNEL) staining using an in situ apoptosis detection kit (TACS, Trevigen). The sections were counterstained with hematoxylin for 30 seconds (pale blue nuclei).

Total plaque size was measured on Movat's pentachrome-stained specimens, macrophage infiltration area was measured on Mac-2-stained specimens, lipid deposition area was measured on Oil Red O-stained specimens, and the number of apoptotic cells was counted from TUNEL-stained specimens.

### Autoradiographic studies and image analysis

The distribution of each tracer in atherosclerotic plaques was determined by dual-tracer autoradiography as described previously [12]. Briefly, cryostat cross sections were exposed to phosphor imaging plates (Fuji Imaging Plate BAS-SR 2025, Fuji Photo Film Co., Ltd., Japan) together with a set of calibrated standards [17]. Co-registration of autoradiographic and histological images was performed as described previously [12]. Regions of interest (ROIs) were manually drawn on atherosclerotic plaques, and ^14^C-FDG or ^99m^Tc-annexin A5 uptake was separately recorded and calculated as percentage injected dose (%ID) and normalized with animal body weight (%ID×kg).

### In vitro studies

THP-1 human monocytic leukemia cell line was obtained from JCRB Cell Bank (Health Science Research Resources Bank, Osaka, Japan). THP-1 cells were cultured in RPMI 1640 medium containing 10% FCS (Sigma, USA) at a density of up to 1×10^6^ cells/ml.

Monocyte chemoattractant protein-1 (MCP-1) mRNA expression level in THP-1 cells was determined by RT-PCR. Briefly, ARBs dissolved in dimethyl sulfoxide were added to 5 ml of the culture medium of 2×10^6^ THP-1 cells at a final concentration of 10 µM. Thirty min after the ARB addition, TNF-α was added to the culture medium at 30 ng/ml and the mixture was incubated for 4–5 hr. PCRs were performed using an SYBR Green I master mix, primers specific for human MCP-1 and glyceraldehyde-3-phosphate dehydrogenase (GAPDH) primers. (5′—3′): MCP-1 sense: caaacccaaactccgaagac; MCP-1 antisense: ttccccaagtctctgtatct; and GAPDH sense: caacgtgtcagtggtggac; GAPDH antisense: gtgtcgctgttgaagtcag. To assess the specific amplification of PCR products, melting curve analysis was performed after the last cycle.

PPARγ activation was assessed using the PPARγ Reporter Assay System (INDIGO Biosciences, Inc. USA) that utilizes non-human mammalian cells engineered to express human PPARγ protein. Following ligand-activation, PPARγ acts to induce expression of the firefly luciferase reporter gene. Briefly, reporter cells were incubated for 24 hr in the medium in the absence or presence of irbesartan or valsartan at the indicated concentrations, and the PPARγ responding element conjugated luciferase expression level was measured.

### Blood pressure measurements

Blood pressure was measured in conscious, restrained female apoE−/− mice by a noninvasive tail-cuff method (BP-2000 Blood Pressure Analysis System; Visitech Systems, Apex, North Carolina, USA). In each recording session, the mice were placed on a heated pad (36°C). The systolic blood pressure of each mouse was measured until blood pressure stabilized, and the average of at least two readings from each mouse was recorded. After recording baseline values of blood pressure and body weight for group allocation, the mice were divided into control and irbesartan-treated groups (16–17 weeks of age, n = 13/group). The control mice were fed a regular research diet (CE-7, Oriental Yeast Co., Ltd.), and the mice in the irbesartan-treated group were fed a diet containing irbesartan (50 mg/kg/day). Blood pressure was measured weekly after the initiation of treatment.

### Statistical analysis

Numerical parameters were expressed as mean ± SD. Un-paired Student's t-test was used to detect significant differences in each parameter between the control and irbesartan treated mice. One-way analysis of variance (ANOVA) followed by the Bonferroni/Dunn test was used to detect significant differences in MCP-1 mRNA expression level among the three treatments. Two-way ANOVA was used to compare the dose-response curves of luciferase activity (PPARγ activation) between the irbesartan and valsartan treatments. The Mann-Whitney U test was used to compare luciferase activity (PPARγ activation) between the treatments at different concentrations and between the two treatments. Linear regression analysis was performed to determine the correlation between the accumulation level of ^14^C-FDG and Mac-2-positive areas and between the accumulation level of ^99m^Tc-annexin A5 and the number of TUNEL-positive cells. Repeated measures ANOVA were used to compare differences in blood pressure between control group and irbesartan-treated group. *P*<0.05 was considered statistically significant.

## Results

### Body weights, blood glucose levels, and plasma lipid profiles

No significant differences were observed in body weight or blood glucose, total cholesterol, LDL-cholesterol, HDL-cholesterol, or triglyceride level between the control and irbesartan-treated groups (Table 1).

**Table 1:** Body Weight, Blood Glucose level and Serum Lipid Profile.

Parameter	Control (n = 11)	Irbesartan (n = 11)	*P* value
Body weight (g)	21.3±0.8	20.9±1.5	*NS*
Blood glucose (mg/dL)	113±22	127±26	*NS*
Total cholesterol (mg/dL)	445±156	457±112	*NS*
LDL cholesterol (mg/dL)	419±157	429±112	*NS*
HDL cholesterol (mg/dL)	7.2±1.3	7.3±1.8	*NS*
Triglyceride (mg/dL)	94.2±33.3	104.4±23.4	*NS*

Data represent mean ± SD (n = 11). NS, not significant.

### Effects of irbesartan on atherosclerotic plaque formation and morphology

**Figure 1:**
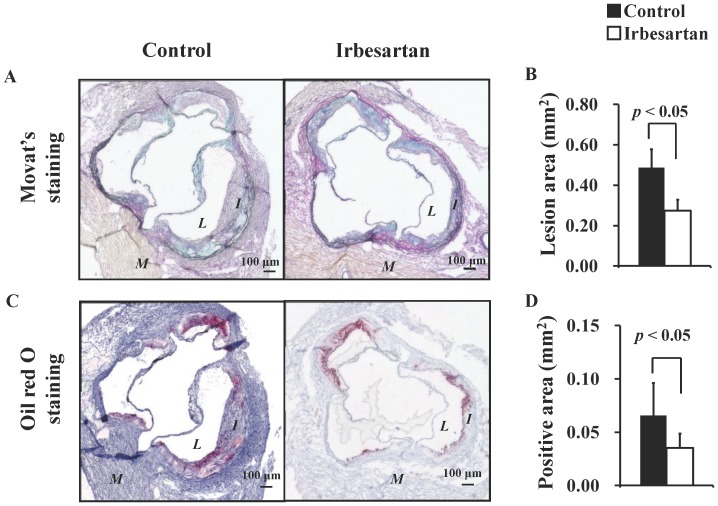
Irbesartan Attenuates Lesion Formation and Lipid Deposition. Movat's pentachrome staining (**A, B**) showed a significant decrease in intimal areas; and Oil Red O staining (**C, D**) showed a significant decrease in lipid deposition area in the irbesartan-treated mice. *M* = myocardium; *I* = intima; *L* = lumen.

**Figure 2:**
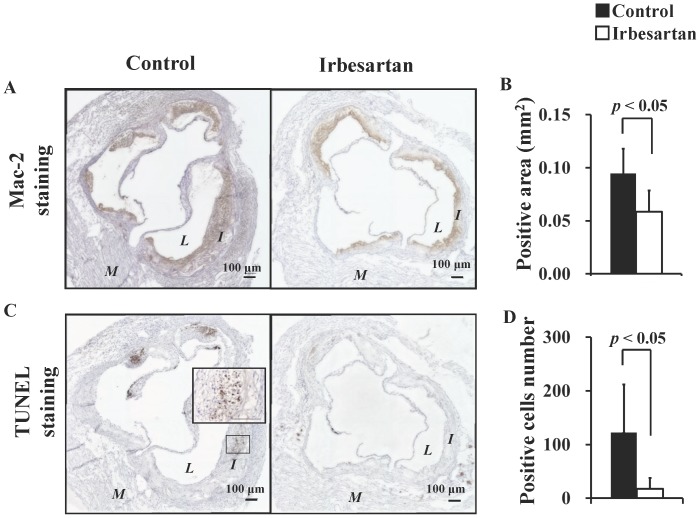
Irbesartan Attenuates Macrophage Infiltration and Apoptosis. Mac-2 staining (**A, B**) showed a significant decrease in macrophage infiltration area, and TUNEL staining (**C, D**) showed a significant decrease in the number of apoptotic cells in the irbesartan-treated mice. The inner box indicates the corresponding magnifications. *M* = myocardium; *I* = intima; *L* = lumen.

Representative photomicrographs of specimens subjected to Movat's pentachrome staining, Oil Red O staining, Mac-2 staining and TUNEL staining are shown Figures 1A, 1C, 2A and 2C, respectively. Plaques of the control mice showed significant intimal thickening with prominent lipid deposition, defused macrophage infiltration, and numerous apoptotic nuclei. In contrast, plaques of the irbesartan-treated mice only showed a thin intima with fewer macrophages, lipid deposits and faint apoptotic nuclei.

Atherosclerotic plaque size assessed by Movat's pentachrome staining demonstrated that irbesartan treatment significantly reduced the plaque formation at aortic root (control mice versus irbesartan-treated mice: 0.49±0.09 mm^2^ versus 0.27±0.05 mm^2^, *P*<0.05) (Fig. 1B). Irbesartan treatment significantly reduced the lipid deposition area (Fig. 1D, 0.07±0.03 mm^2^ versus 0.04±0.01 mm^2^, *P*<0.05), macrophage infiltration area (Fig. 2B, 0.09±0.02 mm^2^ versus 0.06±0.02 mm^2^, *P*<0.05), and the number of apoptotic cells in atherosclerotic plaques (Fig. 2D, 122±89 versus 18±22, *P*<0.05).

### Effects of irbesartan on ^14^C-FDG and ^99m^Tc-annexin A5 levels in the atherosclerotic plaques

**Figure 3:**
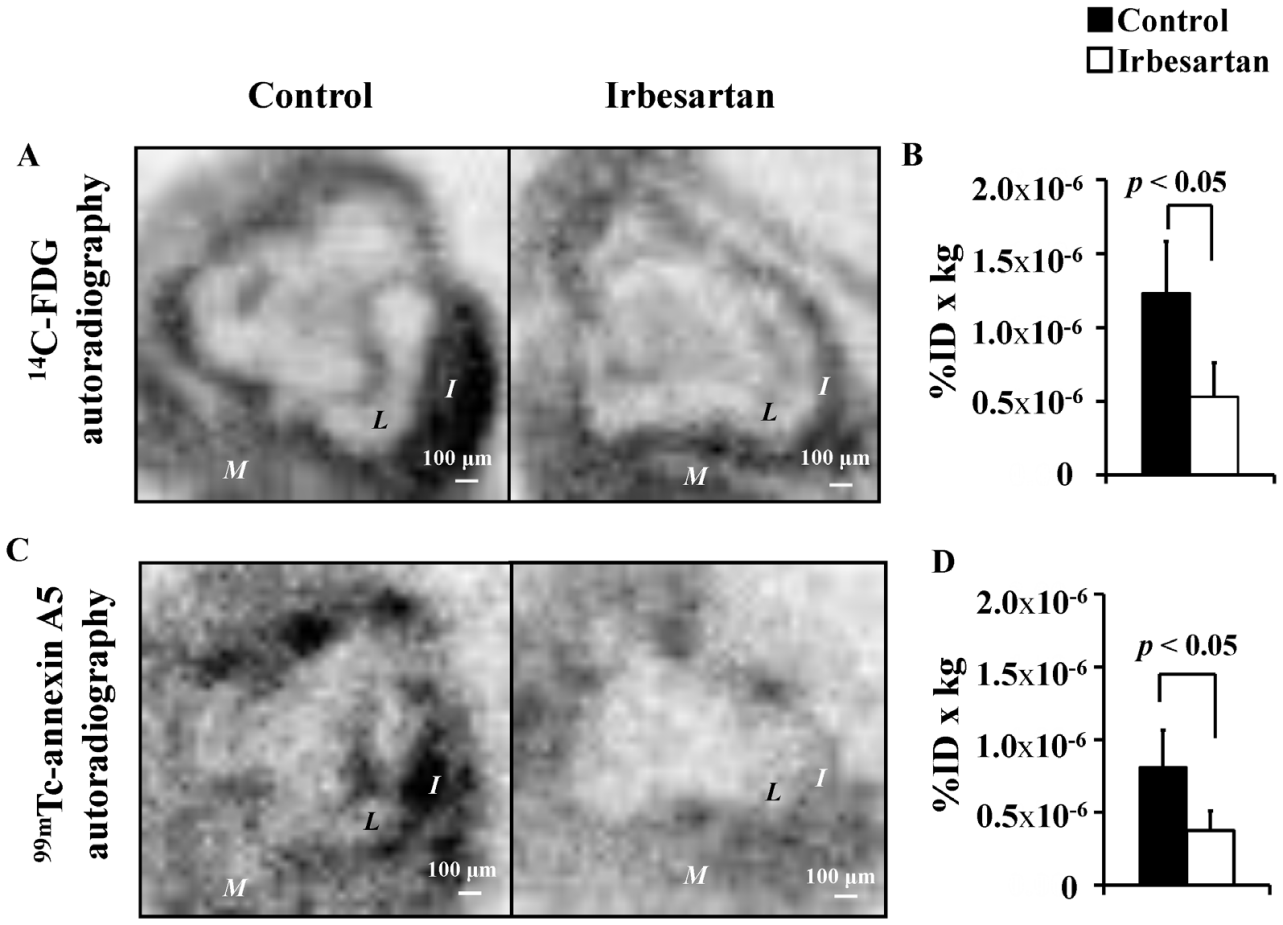
Irbesartan Decreases ^14^C-FDG and ^99m^Tc-annexin A5 Accumulation Levels. The autoradiogram produced by ^14^C signal (**A, B**) showed a significant decrease in ^14^C-FDG uptake levels; and the autoradiogram produced by ^99m^Tc signal (**C, D**) showed a significant decrease in ^99m^Tc-annexin A5 accumulation level in the irbesartan-treated mice. *M* = myocardium; *I* = intima; *L* = lumen.

Representative autoradiographic images of ^14^C-FDG and ^99m^Tc-annexin A5 are shown in Figures 3A and 3C, respectively. In the autoradiographic images and the corresponding photomicrographs of the control mice, the loci of elevated ^14^C-FDG uptake level were consistent with the areas of high macrophage contents, and the loci of elevated ^99m^Tc-annexin A5 level were consistent with areas containing numerous apoptotic nuclei. Compared with the control mice, the irbesartan-treated mice showed suppressed accumulation of ^14^C-FDG and ^99m^Tc-annexin A5 along with decreased macrophage contents and number of apoptotic cells in plaques.

The ^14^C-FDG uptake level in plaques was significantly reduced in the irbesartan-treated mice (Fig. 3B, 1.23±0.35 %ID×kg×10^−6^ versus 0.53±0.23 %ID×kg×10^−6^, *P*<0.05). The ^99m^Tc-annexin A5 level in plaques was also significantly reduced in the irbesartan-treated mice (Fig. 3D, 0.81±0.26 %ID×kg×10^−6^ versus 0.37±0.14 %ID×kg×10^−6^, *P*<0.05).

Positive correlations were observed between the accumulation level of ^14^C-FDG and Mac-2 positive areas (r = 0.69; *P*<0.001) and between the accumulation level of ^99m^Tc-annexin A5 and the number of TUNEL positive cells (r = 0.69; *P*<0.001).

### Suppression of MCP-1 mRNA expression in THP-1 monocytes and PPARγ activation by irbesartan

**Figure 4:**
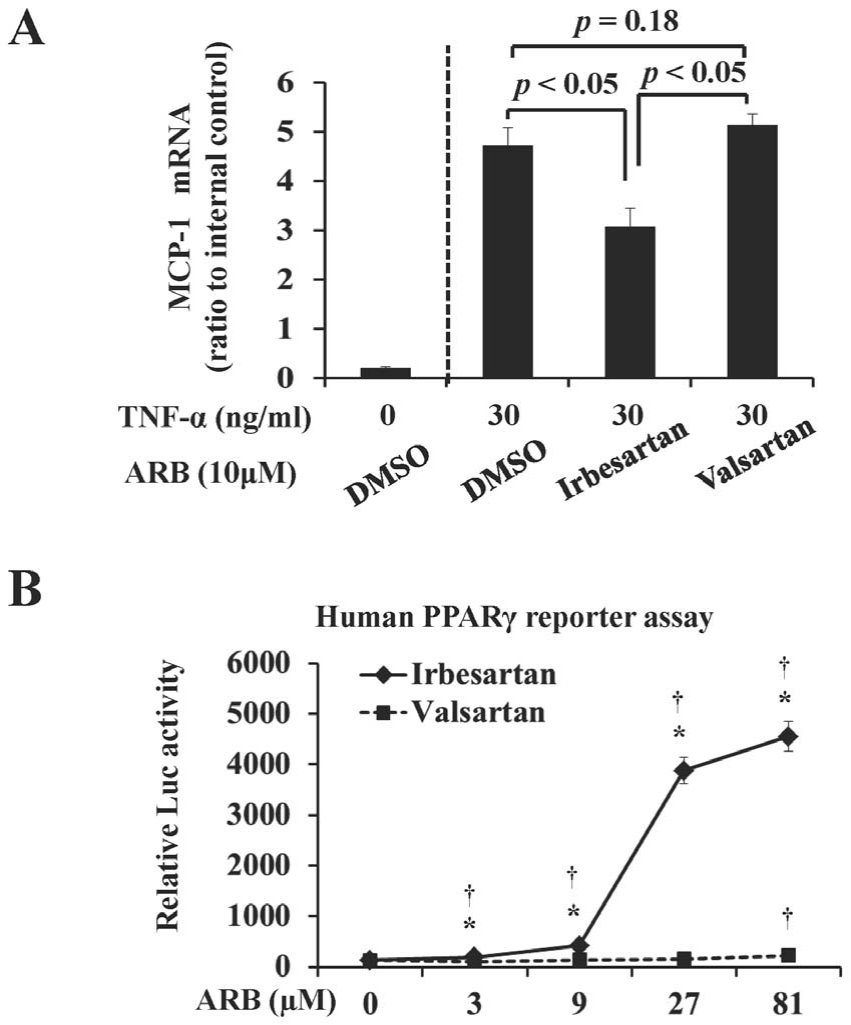
Irbeartan Suppresses MCP-1 mRNA Expression in THP-1 Monocytes and Activates PPARγ in Reporter Cells. Irbesartan significantly suppressed the MCP-1 mRNA expression in THP-1 cells treated with TNF-α (A). Irbesartan significantly activated PPARγ at 3 µM and further activation was observed at higher concentrations, whereas valsartan at its highest concentration (81 µM) only slightly activated PPARγ. Values are means±SD of three separate experiments. *P<0.05 vs valsartan-treated cells; ^†^ *P*<0.05 vs ARB untreated cells.

In THP-1 human monocytic leukemia cells after TNF-α stimulation, irbesartan treatment significantly lowered MCP-1 mRNA expression level (3.06±0.40-fold versus 4.72±0.35-fold, *P*<0.05); however, no significant change was observed in the valsartan-treated cells (5.13±0.23-fold versus 4.72±0.35-fold, *P* = 0.18) (Fig. 4A)

Irbesartan at 3 µM concentration significantly promoted the PPARγ responding element conjugated luciferase expression (Fig. 4B). Moreover, luciferase activity increased in a dose-dependent manner in the cells treated with irbesartan, rather than with valsartan (2-way ANOVA, *p*<0.001). A slight increase in luciferase activity was only observed for 81 µM valsartan (Fig. 4B).

## Discussion

In this study, we demonstrated the following: 1) Irbesartan treatment obviously suppressed atherosclerotic plaque development, lipid deposition, macrophage infiltration, and apoptosis in plaques of apoE^−/−^ mice. In vitro studies showed that irbesartan effectively suppressed the MCP-1 mRNA expression in TNF-α-treated THP-1 cells, and PPARγ activation was observed in the reporter cells treated with irbesartan. 2) The reduced levels of ^14^C-FDG and ^99m^Tc-annexin A5 were observed in plaques of the irbesartan-treated apoE^−/−^ mice. Thus, remissions of inflammation and apoptosis as potential therapeutic effects of irbesartan on atherosclerosis were observed by histochemical examination and molecular imaging using ^14^C-FDG and ^99m^Tc-annexin A5.

Both histochemical examination (Figure 1, Figure 2) and radiotracer imaging findings (Figure 3) confirmed the anti-inflammatory and anti-apoptotic effects of irbesartan in atherosclerotic plaques. The underlying mechanism as the down-regulated inflammatory cytokine expression in macrophage and PPARγ activation in irbesartan treated cells was proved by in vitro experiments (Figure 4).

In humans, the concentration of irbesartan in plasma is in the range from 1.5 to 2.8 mg/ml (4–7 µM) after an oral therapeutic administration of irbesartan (150–300 mg) [18]. Irbesartan at a physiological concentration (3 µM) significantly activated PPARγ in engineered cells expressing the human PPARγ protein (Fig. 4 B). PPARγ activation inhibits inflammatory responses by preventing the activation of nuclear transcription factors, such as nuclear factor-kappa B (NF-κB), and consequently suppresses the production of inflammatory cytokines including MCP-1 [19]. In agreement with this hypothesis, a significant decrease in MCP-1 expression level was also observed in macrophages treated with irbesartan at a similar concentration (Fig. 4 A). This efficacy of irbesartan observed in vitro may contribute to the beneficial effects observed in vivo. A previous in vivo study also confirmed the down-regulation of NF-κB gene transcription and MCP-1 expression in the aorta of irbesartan treated apoE^−/−^ mice [20]. Accordingly, a significant suppression of inflammation after irbesartan administration was confirmed in the present study by histochemical analysis (Fig. 2 A, B) and ^14^C-FDG imaging (Fig. 3 A, B).

**Figure 5:**
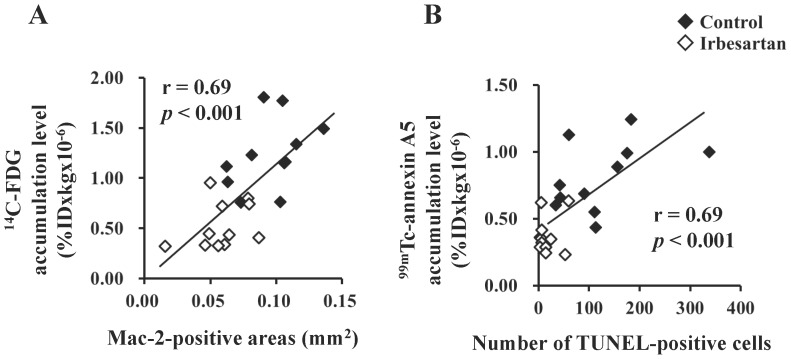
Regression analyses of radiotracer accumulation levels and intraplaque macrophage infiltration/number of apoptotic cells. Positive correlations were observed between the ^14^C-FDG accumulation level and macrophage infiltration (Mac-2-positive areas) (**A**), and between the ^99m^Tc-annexin A5 accumulation level and apoptotic cell number (TUNEL-positive cells) (**B**).

It is recognized and has been demonstrated both by ex vivo studies and in vivo imaging that irbesartan decreases the amount of macrophages in atherosclerosis lesions in mouse models [21]. However, there is a controversy regarding the effect of ARBs on apoptosis. A clinical trial on myocardial infarction demonstrated an increase of 17% in cardiovascular mortality in the case of using losartan (OPTIMAAL), an ARB, compared with the case of using captopril, an angiotensin-converting enzyme inhibitor (ACEI). This may result from unopposed AT2 receptor stimulation, which is associated with enhanced cell apoptosis. By apoptosis imaging with ^99m^Tc-annexin A5, Meijer et al. demonstrated that irbesartan does not induce apoptosis in transient ischemia-reperfusion in humans [22]. A recent animal study indicated that irbesartan can ameliorate the myocardial ischemia/reperfusion injury via down-regulation of apoptosis [23]. Thus, the above mentioned previous findings are inconsistent, and no previous study has directly examined the effects of ARBs on apoptosis in atherosclerotic plaques. In this study, we treated apoE^−/−^ mice with irbesartan and assessed the alteration of apoptosis in plaques by radiotracer imaging and histological examination. A significant reduction in ^99m^Tc-annexin A5 accumulation was observed in the plaques of irbesartan-treated apoE^−/−^ mice, which was associated with the decrease in the number of TUNEL-positive cells (Fig. 5 B). This finding is in agreement with that of our preliminary study using telmisartan, another ARB with PPARγ antagonistic activity [24]. The mechanism underlying the anti-apoptotic effect of irbesartan in plaques may also be related to PPARγ activation. PPARγ activation was reported to contribute to the inhibition of nicotinamide adenine dinucleotide phosphate (NAD(P)H) in the aorta of apoE^−/−^ mice [20], which consequently decreases ROS production and suppresses the apoptosis of vascular cells. The beneficial effect of irbesartan on PPARγ activation has been confirmed in vitro in engineered cells (Fig. 4 B) in this study. However, it is important to perform further studies to verify the effect of ARB on apoptosis in plaques, because ARBs are widely prescribed to patients with cardiovascular risks, and apoptosis greatly contributes to the vulnerability of plaques.

The involvement of PPARγ agonistic activity in the therapeutic effects of irbesartan in apoE^−/−^ mice has also been recently proved by an in vivo study using GW9662, a well-known PPARγ antagonist [25]. Irbesartan can significantly increase the serum concentration of adiponectin (a typical downstream effector of PPARγ) and attenuate chronic inflammation in apoE^−/−^ mice even at a low dose (5 mg/kg/day). The blockade of PPARγ by GW9662 significantly reverses these beneficial effects of irbesartan, which supports the assertion that PPARγ activation is involved in the therapeutic effect of irbesartan [25]. However, in the above-mentioned previous report, the beneficial effects were only examined in the liver and white adipose tissues and not in aortic tissue. Further study should be performed using aortic tissue, and the coadministration of GW9662 and irbesartan should be carried out to prove the involvement of PPARγ agonistic activity in the anti-atherogenic effect of irbesartan.

As for evaluating the therapeutic effects of irbesartan, the beneficial effects of ARBs on plaque morphology have been confirmed by ultrasound [26], cardiac computed tomography (CT), and magnetic resonance imaging (MRI) [27]. However, few challenges in the use of molecular imaging for evaluating the therapeutic effects of ARBs on atherosclerosis remain. The main therapeutic effects of ARBs on atherosclerotic plaques are the suppression of particular plaque composition such as activated macrophages and the suppression of specific molecular process such as cell apoptosis, which may be important and should be considered when evaluating the therapeutic effects of ARBs. With these considerations, we used two molecular functional imaging agents, i.e., ^14^C-FDG and ^99m^Tc-annexin A5, for evaluating the therapeutic effects of irbesartan. As we expected, the present results indicate that the therapeutic effects of irbesartan on atherosclerosis can be quantitatively evaluated using ^14^C-FDG and ^99m^Tc-annexin A5. From the accumulation mechanism of the probes, ^18^F-FDG may be suitable for screening the aortic site with active atheroma and for assessing the suppression of inflammation by ARB treatment, and ^99m^Tc-annexin A5 may be suitable for detecting plaques with enhanced cell apoptosis and assessing the apoptosis suppression [12], [13]. Thus, the above-mentioned findings confirmed the feasibility of ^18^F-FDG and ^99m^Tc-annexin A5 for evaluating the therapeutic effects of ARB on atherosclerosis.

In our previous study, we found the potential anti-apoptotic effect of telmisartan examined by radiotracer imaging, but we did not confirm such an effect by histological examination [24]. In this study, we demonstrated the close relationship between histological findings and radiotracer imaging findings, which proves the rationale for using nuclear imaging to evaluate the beneficial effects of ARBs on atherosclerosis. At the same time, irbesartan activates PPARγ in reporter cells, which indicates the underlying mechanism, and partly explains why controversy exists over the effect of ARBs on apoptosis.

The effects of irbesartan on blood pressure should be noted. Unfortunately, we did not monitor the changes in blood pressure during the treatment period. However, the blood pressure-lowering effect of irbesartan was shown by our preliminary study (Figure S1). Soon after the initiation of treatment, a notable decrease in systolic blood pressure was observed in the irbesartan-treated mice whose blood pressure was significantly lower than that in the control. The blood pressure was decreased by about 20 mmHg following irbesartan treatment at a dose of 50 mg/kg/day, in agreement with the finding of a previous study, which used a similar treatment protocol [28]. These effects of irbesartan on blood pressure may contribute to its anti-atherosclerotic effects. However, atenolol, a beta receptor blocker, exerts blood pressure lowering effects comparable to irbesartan but not retards the progression of atherosclerosis [29]. Clinical study also indicated that the anti-atherogeneic effect of ARBs is beyond that from the decrease in blood pressure [30].

## Conclusions

We observed remissions of inflammation and apoptosis in atherosclerotic plaques of apoE^−/−^ mice treated with irbesartan. Our in vitro studies also indicated that irbesartan suppressed atherogenesis by attenuating inflammatory cytokine expression in macrophages through PPARγ activation. The suppression of macrophage infiltration and apoptosis is the main functional outcome of irbesartan treatment, which should be evaluated. Such effects of irbesartan on atherosclerosis can be non-invasively imaged by molecular imaging using ^18^F-FDG and ^99m^Tc-annexin A5.

## Supporting Information

Figure S1 Time course of systolic blood pressure in control and irbesartan-treated apoE−/− mice. The systolic blood pressure in the control mice (•) was significantly higher than that in irbesartan-treated mice (○). Values are means ± SD.
(TIFF)
