# Supplementary figures and images for: Suppressive Effects of Irbesartan on Inflammation and Apoptosis in Atherosclerotic Plaques of apoE−/− Mice: Molecular Imaging with 14C-FDG and 99mTc-Annexin A5
Source: PLoS One. 2014 Feb 19;9(2):e89338. doi: 10.1371/journal.pone.0089338 (PMC3929710; doi:10.1371/journal.pone.0089338)

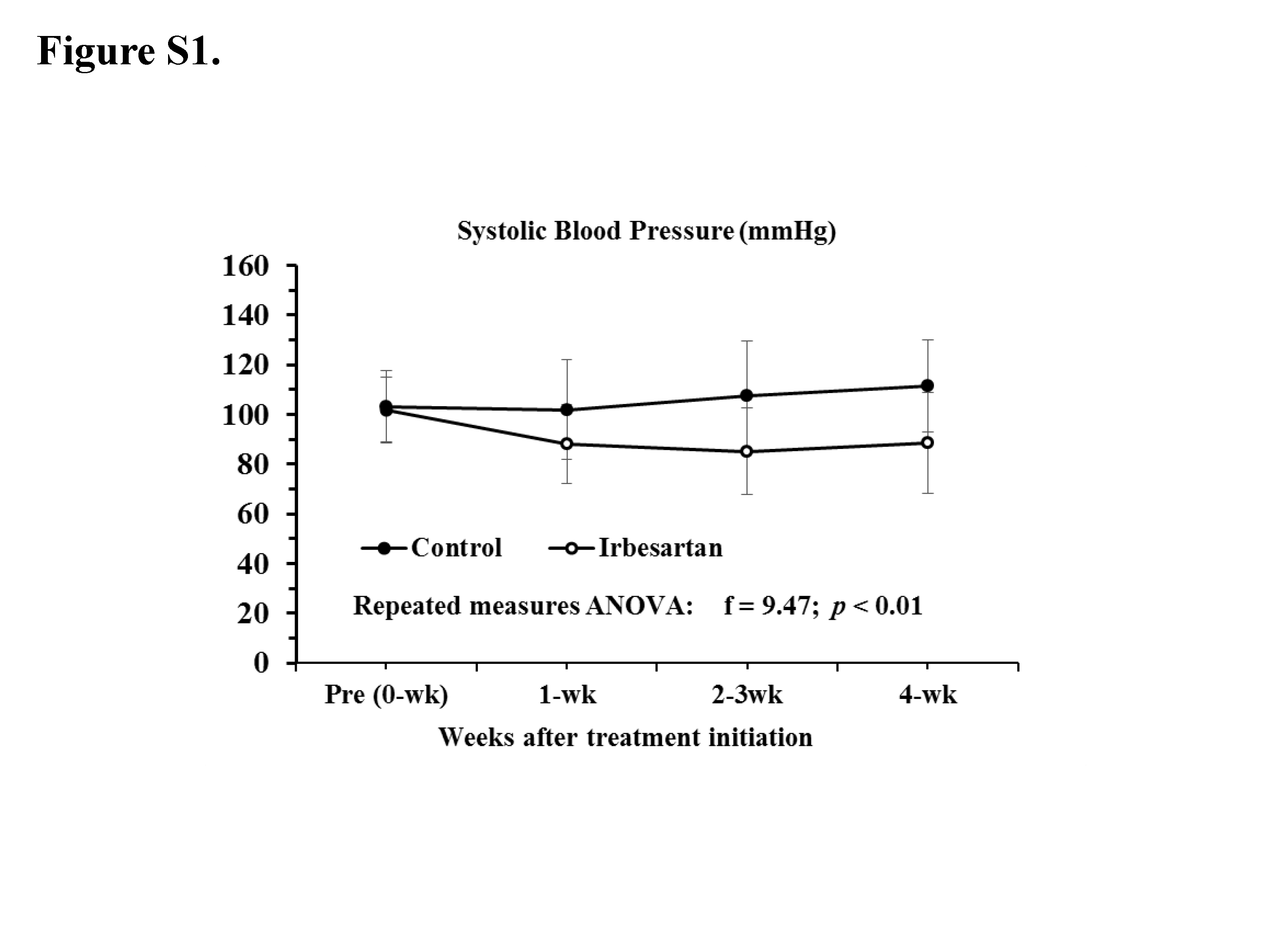

Supplement: Figure S1 — Time course of systolic blood pressure in control and irbesartan-treated apoE−/− mice. The systolic blood pressure in the control mice (•) was significantly higher than that in irbesartan-treated mice (○). Values are means ± SD. (TIFF) [file pone.0089338.s001.tiff]
